# Ohio Dental College - Commencement

**Published:** 1874-03

**Authors:** 


### OHIO DENTAL COLLEGE—COMMENCEMENT.

The twenty-eighth annual commencement of this institution was held on the evening of Feb'y 24th, 1874, in the lecture hall of the College.

The exercises were opened by reading the Scriptures, remarks and prayer by Rev. Dr. Boynton, after which the annual address was delivered by Dr. Geo. W. Keely, of Oxford, O., at the conclusion of which a brief address was made and the degrees conferred by Dr. Jas Taylor, President of the Board of Trustees. The custom, in this institution, of giving a bible with each diploma was observed.

An address on behalf of the graduating class was delivered by Geo. H. Mosher.

The degree of D. D. S. was conferred upon the following gentlemen: Geo. H. Mosher, Jackson, Mich.; Louis L. Dunbar, Cincinnati; William Hare, Cincinnati; W. D. Dismukes, Nashville, Tenn.; J. C. Oldham, Springfield, O.; R. W. Morris, Santa Barbara, Cal.; S. W. Moores, Maysville, Ky.

The session has been a pleasant and, we trust, a profitable one, though the class was small.

